# Microalgae, a Boring Bivalve and a Coral—A Newly Described Association Between Two Coral Reef Bioeroders Within Their Coral Host

**DOI:** 10.1093/iob/obaa035

**Published:** 2020-10-30

**Authors:** A J Fordyce, T D Ainsworth, W Leggat

**Affiliations:** School of Environmental and Life Sciences, University of Newcastle, Ourimbah, NSW 2258, Australia; School of Biological, Earth and Environmental Sciences, University of New South Wales, Sydney, NSW 2052, Australia; School of Environmental and Life Sciences, University of Newcastle, Ourimbah, NSW 2258, Australia

## Abstract

Bioeroding organisms play an important part in shaping structural complexity and carbonate budgets on coral reefs. Species interactions between various bioeroders are an important area of study, as these interactions can affect net rates of bioerosion within a community and mediate how bioeroders respond to environmental change. Here we test the hypothesis that the biomass of endolithic bioeroding microalgae is positively associated with the presence of a macroboring bivalve. We compared the biomass and chlorophyll concentrations of microendolithic biofilms in branches of the coral *Isopora palifera* (Lamarck, 1816) that were or were not inhabited by a macroboring bivalve. Those branches with a macroborer present hosted ∼80% higher microbial biomass compared to adjacent branches from the same coral with no macroborer. Increased concentrations of chlorophyll *b* indicated that this was partly due to a greater abundance of green microalgae. This newly described association has important implications for the coral host as both the bivalve and the microalgae have been hypothesized as symbiotic.

## Introduction

The process of bioerosion (the decay, degradation, or dissolution of calcium carbonate by living organisms) modulates multiple aspects of reef ecological function. Bioeroders modify the structural complexity of reefs at scales from microns to meters (Glynn and Manzello 2015; Davidson et al. 2018; Roff et al. 2019), which influences coral reef herbivory (Vergés et al. 2011), coral and fish larval settlement (Coker et al. 2012; Kegler et al. 2017), and a reef’s economic value ( Graham and Nash 2013; see also Schönberg et al. 2017 ). Bioeroders can be characterized into guilds depending upon their habitat (epilithic or endolithic), size (micro and macro), and mechanism of bioerosion (Schönberg et al. 2017), with both mechanical and chemical mechanisms being employed by various guilds. Some bioeroding taxa such as excavating parrotfish, scraping urchins, and boring sponges physically break down carbonate substrates and produce coarse carbonate sediments on a reef. Sediment turnover is important in maintaining sediment porosity and permeability, which affects the productivity of coral reef sands (Miyajima et al. 2001; Santos et al. 2010). Other bioeroders dissolve, rather than degrade, carbonate substrates and so affect reef carbonate budgets by modulating the availability of dissolved inorganic carbon (Perry et al. 2014). Bioerosion, therefore, affects both reef growth and productivity.

Importantly, there is evidence that ecological interactions between bioeroders are capable of enhancing or suppressing overall rates of erosion on a coral reef. As such these are key considerations when seeking to understand how bioerosion is shaping a reef environment. For example, grazing urchins may target endolithic microalgae (i.e., living within the rock) as a food source and scrape the substrate as they feed (Chazottes et al. 1995). Despite this, predation by urchins ultimately increases bioerosion by microendoliths due to the increased light field in the substrate which extends the compensation depth for algal colonization (Chazottes et al. 1995; Tribollet and Golubic 2005). Parrotfish and urchin grazing have also been found to control recruitment and succession in endolithic macroborer communities in Kenya, resulting in reduced overall rates of macrobioerosion on the reef (Carreiro-Silva and McClanahan 2012). These inter-guild interactions between microborers, macroborers, and grazers can take the form of feedback loops in which one guild might enhance or suppress bioerosion by a different guild (Carreiro-Silva and McClanahan 2012; Schönberg et al. 2017). Environmental change can disrupt the balance between these species and alter pathways in these loops, leading to shifts in ecosystem bioerosion (Perry et al. 2014; Schönberg et al. 2017). Therefore, we can benefit from a greater understanding of the number and structure of bioeroder interactions within an ecological web.

Recently, Rice et al. (2020) identified an interaction between two bioeroding organisms, excavating parrotfish and endolithic lithophagine mussels inhabiting the skeleton of live massive *Porites* spp. corals. Similar to the results presented by Rotjan and Lewis (2005), the authors identified a positive relationship between the density of macroborers in a live coral and the frequency of parrotfish bite scars on the same colony. Both studies suggested that targeted feeding by parrotfish on macroborers drove this association, and Rice et al. (2020) went further to hypothesize that this interaction might be partly mediated by endolithic microalgae living alongside the bivalves inside the coral skeleton. Excretion by the bivalve, which underlies its putative beneficial role to corals (Mokady et al. 1998), could effectively fertilize the surrounding skeleton and so increase the abundance of endolithic microalgae. The blue mussel, *Mytilus edulis*, has been shown to enrich sediment porewater through the biodeposition of ammonium and phosphates, boosting the growth of co-occurring seagrass (Reusch et al. 1994). By enriching the endolithic habitat of a coral colony with waste products, bivalves are potentially increasing the abundance of endolithic algae which increases the nutritional value of that patch of coral colony for a grazing parrotfish. This is especially true for “microphagous” parrotfish, a termed used to describe their preferential feeding habits on areas abundant in microalgae (Bruggemann et al. 1994; Clements et al. 2016).

Here we investigate the potential for this undescribed association occurring inside the skeleton of a living coral, with the potential to influence coral health and skeletal integrity. We tested the hypothesis of Rice et al. (2020) that the presence of macroborers is associated with an increased biomass of microalgae in the endolithic habitat of *Isopora palifera* (Lamarck, 1816) coral colonies.

## Methods

### Initial observations

**Fig. 1:**
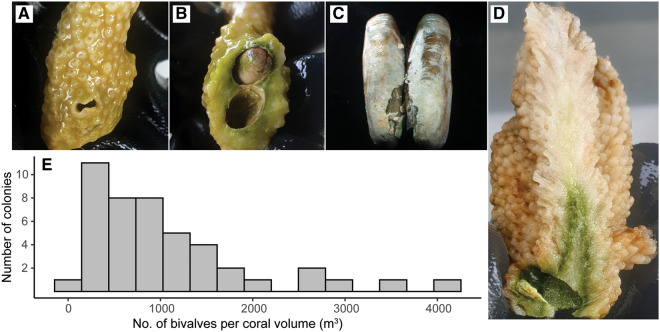
(**a**) External view of a borehole made by a lithophagine bivalve. Note the darker coral tissue pigmentation around the bore hole. (**B**) Internal view of the same borehole. Note the thick green band surrounding the bore hole. (**C**) A lithophagine mussel removed from the coral skeleton. (**D**) longitudinal cross-section of a coral skeleton with a lithophagine bore hole. (**E**) Histogram of macroborer density defined as number of individuals per approximate cubic meter of coral skeleton (*n* = 45 colonies surveyed).

In October 2019, we observed that the presence of bore holes made by lithophagine bivalves (Fig. 1A–C) were surrounded by a dense green “halo” inside the skeleton of *I. palifera* (Fig. 1B and D). This species of coral has been previously recognized as being frequently infested with lithophagine bivalves (Kleemann 1980). *Isopora palifera* exhibits variable morphology that can be predominantly encrusting or sub-massive with thick, columnar, or plate-like branches (Veron and Stafford-Smith 2000). In the Heron Island reef lagoon, this species is primarily sub-massive with columnar branches, forming stand-alone branching colonies. The green patches within the skeleton were at times macroscopically visible through the live coral tissue as a green hue (Fig. 1B). In January 2020, we conducted a survey of the Heron Island reef lagoon (0.5–2 m depth; 23.4423° S, 151.9148° E), at low tide, to assess the prevalence of lithophagine macroborer boreholes within *I. palifera* colonies, identified by the conspicuous figure-eight borehole shapes (Kleemann 1980) (Fig. 1A). We haphazardly chose a direction across the reef flat, walked 15 m, and then surveyed the nearest *I. palifera* colony for the presence of lithophagid boreholes. We then chose another random direction and repeated this step until 45 colonies were surveyed (15 m was chosen to reduce the chances of surveyed colonies being clonal). The maximum height, width, and depth of each colony were measured using a ruler as a coarse estimate of colony volume against which we could estimate macroborer density as the number of individuals per cubic meter (Fig. 1E).

### Sample collection

Two branches from each of 20 colonies (*n* = 40) were collected using a hammer and chisel in January 2020: one branch inhabited by a single lithophagid macroborer and one without any macroborer boreholes. Each coral colony was selected haphazardly and was a minimum of 15-m apart. When selecting the two branches, we chose two that were adjacent to each other on the horizontal plane and that did not visibly experience significantly different light environments. We hypothesized that microendolithic biomass would be affected by self-shading amongst the branches of the colony, although knowledge of intra-colony variation in endolithic biomass is currently limited. This decision was made to minimize bias from intra-colony variation in endolithic biomass due to variable light environments. The branches were collected under permit G18/41124.1 and did not exceed 7 cm in length or 4 cm in diameter.

### Sample processing

To minimize contamination by photosymbiotic dinoflagellates living in the coral tissue, coral tissue was removed using a compressed air gun (Ozito, Australia) attached to a 15 L aluminum SCUBA cylinder. Following this, the macroborers were also removed from those samples they inhabited. This was achieved by cracking open the skeleton using bone cutters and using forceps to remove the bivalve. In the process of removing the bivalve, each sample was also inspected for the presence of other macroborers (e.g., polychaetes). After airbrushing, the skeletal volume of each fragment was measured using Archimedean principles as per the buoyant weight technique (Jokiel 1978). For the split-open samples from which macroborers were removed, the pieces of the skeleton were weighed together in the weighing basket, following the apparatus described by Jokiel (1978). Seawater was supplied from the reef-flat and its density calculated as 1.0245 g cm^−3^, using a stainless steel double-ended snap-bolt as a reference object. The density of the reference object was calculated by comparing its weight in air and distilled water, and this value was used to then calculate seawater density. For every 0.1°C change in temperature, seawater density was re-calculated.

Samples were weighed while suspended in seawater. After, we back-calculated coral dry weight using the density of pure aragonite which is 2.947 g cm^−3^ (Jokiel 1978). This was used in lieu of a published estimate for the specific skeletal density (termed micro-density) of *I. palifera*. The equation to back-calculate dry weight was:

$${\mathrm{Branch}}_{\mathrm{Dry}\;\mathrm{mass}}={\mathrm{Branch}}_{\mathrm{SW}\;\mathrm{mass}}/(1-\left(\frac{{\mathrm{SW}}_{\mathrm{dens}}}{2.947}\right))$$

Then, sample weight in seawater is subtracted from sample dry weight and divided by the density of seawater to produce an estimate of the volume of each coral branch.

After samples were weighed, they were dissolved in acid following modified methods from Fine and Loya (2002). Samples were dissolved in sequential washes using 1.6 M hydrochloric acid within 50 mL plastic centrifuge tubes. Between each acid change, the samples were centrifuged at 3856 × g and 4°C for 10 min, in order to separate out the skeletal organic matrix of the coral host. The reacted acid (now calcium chloride), including the organic matrix, was decanted and fresh acid added. The resulting microendolithic pellet was washed twice with 0.22 µm filtered seawater to remove excess acid, was resuspended in 20 mL of filtered seawater, and then homogenized by a combination of vigorous shaking and vortexing for up to a minute to break apart the pellet. A 10 mL syringe was used to transfer half of the homogenized endolithic mixture of each sample to a paired centrifuge tube.

### Ash free dry weight

About 10 mL of each endolithic sample was first centrifuged and a 10 mL syringe was then used to remove 9 mL of the clear supernatant, the remaining pellet was dislodged by gentle shaking and roughly resuspended in the remaining 1 mL of supernatant. The solution was then poured into a sterilized, pre-burned crucible; any particulate still in the tube was washed into the same crucible using filtered seawater. Crucibles were dried in an oven at 70°C for 18 h, leaving a combination of dried organic matter and inorganic minerals. These dried crucibles were weighed on a four decimal place balance (Ohaus, Parsippany, NJ, USA) and then placed in a muffle furnace at 550°C for 4 h to burn off all organic matter (Reyes-Nivia et al. 2013). After cooling, they were weighed again and the biomass of endolithic phototrophs calculated as the reduction in mass as a result of burning (i.e., ash-free dry weight [AFDW]). This was normalized to half the branch volume as the homogenized endolithic sample was split prior to chlorophyll and biomass analysis. AFDW is measured in g cm^−3^.

### Chlorophyll concentrations

The remaining 10 mL of each sample was centrifuged to produce a concentrated endolithic pellet. All of the supernatants were carefully removed and the pellet resuspended in 10 mL of 90% acetone to extract chlorophyll (Ritchie 2008). After adding the acetone, samples were vortexed for 30 s and placed in an ultra-sonicator (Unisonics, NSW, Australia) for 20 min to break apart cell walls. Suspensions were then vortexed for another 30 s and left to extract for 24 h at 4°C in the dark. Samples were then centrifuged using the same settings as above to produce a clear green supernatant containing dissolved chlorophyll, and each sample was pipetted in triplicate into a microplate to be analyzed using a spectrophotometer (Spectrostar Nano, BMG Labtech, Australia). A paired 90% acetone blank solution was measured between each sample in the microplate read direction. The blank-corrected raw absorbance values were converted to µg cm^−3^ using the quadrichroic spectrophotometric equations of Ritchie (2008) that measure chlorophylls *a, b, c*, and *d*. We selected this method in favor of trichroic equations (e.g., Jeffrey and Humphrey 1975) because chlorophyll *d*-containing cyanobacteria has been previously identified from endolithic habitats at this location (Behrendt et al. 2011) and not accounting for the presence of chlorophyll *d* can lead to overestimates of chlorophylls *a* and *b* (Ritchie 2008). The decalcification of the samples using HCl acidified chlorophyll *a* in our samples to pheophytin *a*, which lowers absorption at 664 nm and broadens the absorption peak, leading to the underestimation of chlorophyll *a* by spectrophotometry (Ritchie 2008). Nonetheless, this method still produces results that are well correlated with techniques such as high-performance liquid chromatography (Grinham et al. 2007; Ritchie 2008). To normalize concentrations to branch size, the triplicate sub-samples were first averaged to give a sample-wise concentration in µg cm^−3^ and then multiplied by ten to give an estimate of the total weight of chlorophyll in each 10 mL sample, in µg. These values were then divided by half the fragment volume to give a chlorophyll concentration per cubic centimeter of coral skeleton.

### Statistics

All statistics were performed using R version 3.6.0 (R Core Team 2019). We used paired two-tailed *t*-tests to analyze the five parameters (biomass, chlorophylls *a*–*d*) of samples with and without macroboring bivalves. To test the assumption that the dependent variables were normally distributed, we used a combination of Shapiro–Wilk tests and Q–Q plots. Potential outliers were identified using a Cook’s distance of 4/*n* (Cook 1977). When present, we compared the model outcomes with and without the presence of the outlier(s) and in all cases they had no effect upon the results of the *t*-test. All chlorophyll concentration tests violated the assumption of normality. Log-transforming the data did not address these violations, so we used Wilcoxon signed-rank tests to analyze these variables. All reported values are mean ± S.E.

## Results

**Fig. 2:**
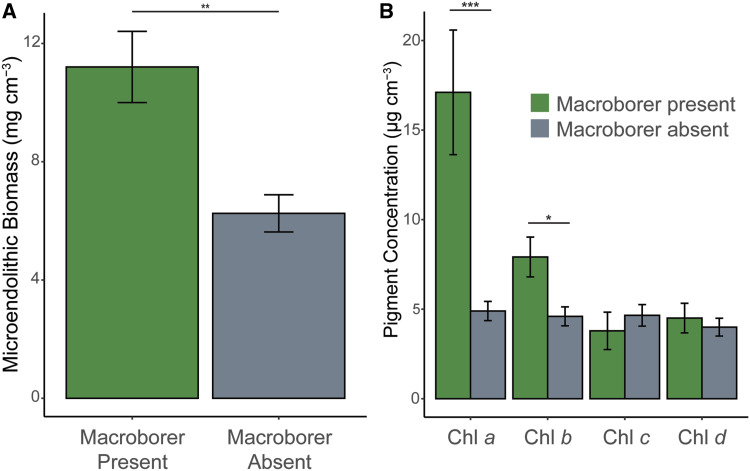
Results from analyses of branches with (green) or without (gray) a macroborer present. (**A**) Barplot of AFDW microbial biomass of endolithic community. (**B**) Barplots of concentrations of chlorophylls *a*–*d*. Bars represent mean ± S.E. *, **, and *** denote significant differences of relative to α thresholds of 0.05, 0.01, and 0.001, respectively.

We surveyed 45 *I. palifera* colonies on the Heron Island reef flat in January 2020 and recorded a median density of 832 lithophagine boreholes per cubic meter of coral (Fig. 1E). When comparing coral branches with and without a bivalve macroborer, we found that branches previously inhabited by a macroborer had an endolithic microbial biomass that was significantly greater than that recorded for branches without a macroborer (*t*_19_ = 3.220, *P* = 0.005; Fig. 2A). The mean chlorophyll *a* concentration in branches where a macroborer was present was significantly greater than the concentration recorded for branches without a macroborer (*z* = −4.001, *P* < 0.001; Fig. 2B). Similarly, the mean chlorophyll *b* concentration measured from the endolithic microbiome in branches with a bivalve was significantly greater than for branches without a macroborer (*z* = −2.383, *P* = 0.017; Fig. 2B). The concentrations of chlorophylls *c* and *d* did not differ significantly between groups (chlorophyll *c*: *z* = −1.658, *P* = 0.097; chlorophyll *d*: *z* = −0.018, *P* = 0.985).

## Discussion

Lithophagine bivalve macroborers, identified by the figure-eight shape of their boreholes (Kleemann 1980), were present in all but one of the surveyed *I. palifera* colonies on the Heron Island reef flat (Fig. 1E). The median density of boreholes per cubic meter of coral was slightly lower than those recorded on previous surveys of date mussel density in massive *Porites* spp. (Rotjan and Lewis 2005; Rice et al. 2020). However, our method for approximating coral volume might be expected to overestimate the true volume of substrate available for macroboring. In the *I. palifera* branches inhabited by a macroboring bivalve, the microendolithic biomass was almost double that of adjacent, uninhabited branches from the same colony (Fig. 2A). Additionally, the concentrations of chlorophylls *a* and *b* were ∼4- and 2-fold greater in the presence of a macroborer (Fig. 2B). Chlorophyll *b* is the primary accessory pigment found in microendolithic green algae, such as *Ostreobium* spp., which commonly dominate the microboring communities of coral skeletons (Marcelino and Verbruggen 2016; del Campo et al. 2017; Ricci et al. 2019; Pernice et al. 2020). Our data, therefore, suggest that the increased microbial biomass was in part due to a higher abundance of green microalgae in the skeleton. The data also indicate the presence of chlorophyll *d*-containing cyanobacteria within our *I. palifera* samples. The only recorded genus of alga known to use chlorophyll *d* is *Acaryochloris* (Larkum and Kühl 2005), which has been identified previously from endolithic habitats under crustose coralline algae at this location (Behrendt et al. 2011).

We have found evidence of a positive association between the presence of a lithophagine bivalve macroborer and the biomass of endolithic microalgae, within an *I. palifera* coral host. Both Rotjan and Lewis (2005) and Rice et al. (2020) found that parrotfish bite frequency on a coral colony was correlated with the density of resident macroboring bivalves. All authors suggested that these relationships could reflect targeted feeding of parrotfish on nutrient-rich macroborers, as Rotjan and Lewis (2005) found no difference in the nutritional quality of overlying coral tissue. Rice et al. (2020) went on to hypothesize that it may also be partly mediated by microendolithic algae which live alongside the macroborer in the coral skeleton. Our results lend credence to this hypothesis. Higher microalgal biomass in the skeleton concomitant with macroborer infestation would increase the nutritional value of a particular patch of coral. This is especially true if the parrotfish species show preferential feeding on microalgae (Bruggemann et al. 1994; Clements et al. 2016) and/or is omnivorous (Bellwood and Choat 1990). Similarly, the bivalve–microendolith association could help explain the results presented by Simon-Blecher et al. (1996). They recorded higher chlorophyll fluorescence in healthy coral tissue adjacent to a lithophagine borehole in the coral *Goniastrea* sp. compared to tissue without a borehole next to it. They suggest that this might reflect nitrogen-enrichment of coral tissue through bivalve excretion (Simon-Blecher et al. 1996). However, Rotjan and Lewis (2005) found no difference in coral tissue nitrogen content whether next to or away from a borehole. Instead the stronger fluorescence signal may have been due to the microendolithic “halo,” as described here (Fig. 1), beneath the coral tissue. Fine et al. (2005) have previously shown that endolithic algae beneath the tissue of the coral *Oculina patagonica* can influence chlorophyll fluorescence measured in the coral tissue.

**Fig. 3:**
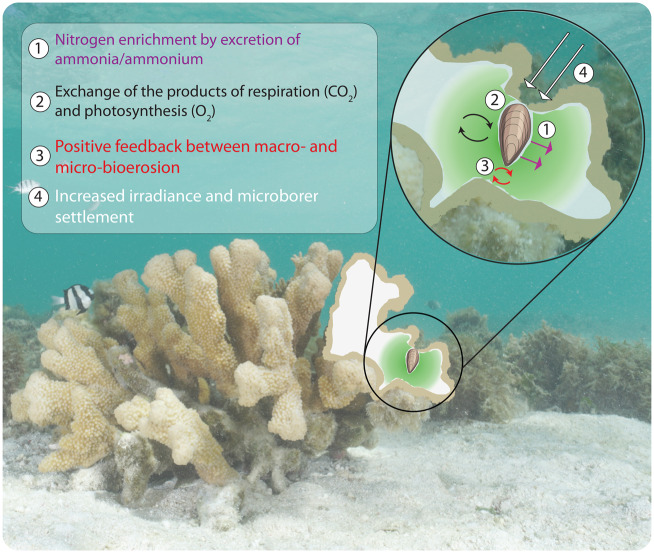
Conceptual diagram illustrating the hypothetical mechanisms driving the relationship described here. (**1**) Biodeposition of nitrogenous compounds through bivalve excretion enhances microalgal growth. (**2**) Exchange of the products of photosynthesis and respiration for mutual benefit. (**3**) Each bioeroder reduces the energetic cost of boring for its skeletal co-habitant. (**4**) The open borehole increases irradiance for and promotes settlement of microalgal endoliths.

There are several possible mechanisms driving the association between macroboring bivalves and algal microendoliths inside the skeleton of *I. palifera*. First and foremost is the potential for fertilization of the endolithic microhabitat by excretion of nitrogenous waste from the macroboring bivalve. This dynamic underlies the hypothetical mutualism between corals and boring bivalves (Mokady et al. 1998) and occurs in seagrass beds where infaunal blue mussels, *M. edulis*, enhance the growth of *Zostera marina* by fecal biodeposition (Reusch et al. 1994). Additionally, artificial enriching the endolithic habitat with nitrogenous inorganic matter increases colonization and bioerosion by microendoliths in the shells of giant clams *Strombus gigas* (Carreiro-Silva et al. 2005, 2009, 2012). It is also possible that CO_2_ produced by bivalve respiration diffuses into the skeleton and enhances daytime microbial photosynthesis. This could be coupled with oxygen produced by photosynthesis diffusing into the macroborer burrow; endolithic algae have been previously shown to cause skeletal porewater to become supersaturated with respect to oxygen (Kühl et al. 2008). Finally, there is evidence that photoassimilates produced by endolithic algae are a source of sugars for the coral host (Schlichter et al. 1995; Fine and Loya 2002; Sangsawang et al. 2017) and this may also be the case for lithophagine bivalves. Taken together, this is suggestive of metabolic exchange between endolithic bivalves and microalgae in the form of series of positive feedback loops (Fig. 3). The limited diffusion of metabolic waste products through the skeleton may also explain the shape of the green “halos” around macroborer boreholes. This has important implications for the spatial extent of this association within a coral colony.

Bioerosion by microendolithic algae can promote colonization by macroborers such as polychaetes and sponges, by weakening substrates and thereby reducing the energetic cost of macroborer colonization (Che et al. 1996; Schönberg et al. 2017). Equally the secretion of an acidic mucus by the macroborer, which is the primary form of chemical erosion in boring bivalves (Kleemann 1996), weakens skeletal matrices (Scott and Risk 1988), and so may reduce the energetic cost of boring for microendoliths. Additionally, the presence of an external opening on the coral surface may increase the endolithic light field and promote more settlement by microendoliths that colonize new substrates from the water column as opposed to from neighboring substrates (Massé et al. 2018) (Fig. 3). Therefore both members of this association have the capacity to promote colonization and bioerosion by each other. In the freshwater bivalve *Lignopholas fluminalis*, cooperation with co-occurring microorganisms was found to promote the bioerosion of silicate siltstone (Daval et al. 2020). The association described in this study may therefore be maintained through metabolic exchange and/or by the combined weakening of the coral skeleton (Fig. 3). It is beyond the scope of this study to state how the relationship is first established. It is possible that the initial trigger is macroborer settlement which enhances growth in the already present microendolith community, which then serves to reduce the energetic cost of burrowing by the bivalve. In this vein, pre-existing microendolithic biomass (i.e., before infestation by a macroborer) might be an important factor affecting settlement success (i.e., recruitment) in bivalves.

The bivalve–microendolith association described here is comparable to the relationship between macroboring polychaetes and microendoliths, wherein each guild promotes bioerosion by the other (Che et al. 1996; Schönberg et al. 2017). These inter-guild relationships affect overall rates of bioerosion on a reef through the “bioerosion loop” (Carreiro-Silva and McClanahan 2012; Schönberg et al. 2017). While it is not clear how this relationship between boring bivalves and microendoliths affects net bioerosion, it does have some interesting implications for the health of the coral host. Both lithophagine bivalves and endolithic microalgae have been previously independently proposed as symbiotic to their host coral (Mokady et al. 1998; del Campo et al. 2017). Describing and understanding these multi-species interactions are a promising area for discovery and continues to be an important step in understanding the role of bioeroders on coral reefs.

## Supplementary Material

obaa035_Supplementary_Data

## References

[obaa035-B1] Behrendt L, Larkum AW, Norman A, Qvortrup K, Chen M, Ralph P, Sørensen SJ, Trampe E, Kühl M. 2011 Endolithic chlorophyll d-containing phototrophs. ISME J 5:1072–6. 21160540 10.1038/ismej.2010.195 PMC3131860

[obaa035-B2] Bellwood DR, Choat JH. 1990 A functional analysis of grazing in parrotfishes (family Scaridae): the ecological implications. Environ Biol Fishes 28:189–214.

[obaa035-B3] Bruggemann H, van Oppen M, Breeman AM. 1994 Foraging by the stoplight parrotfish *Sparisoma viride*. I. Food selection in different, socially determined habitats. Marine Ecol Progr Ser 106:41–55.

[obaa035-B4] Carreiro-Silva M, Kiene W, Golubic S, McClanahan T. 2012 Phosphorus and nitrogen effects on microbial euendolithic communities and their bioerosion rates. Marine Pollut Bull 64:602–13. 10.1016/j.marpolbul.2011.12.013 22240204

[obaa035-B5] Carreiro-Silva M, McClanahan T. 2012 Macrobioerosion of dead branching Porites, 4 and 6 years after coral mass mortality. Marine Ecol Progr Ser 458:103–22.

[obaa035-B6] Carreiro-Silva M, McClanahan TR, Kiene WE. 2005 The role of inorganic nutrients and herbivory in controlling microbioerosion of carbonate substratum. Coral Reefs 24:214–21.

[obaa035-B7] Carreiro-Silva M, McClanahan TR, Kiene WE. 2009 Effects of inorganic nutrients and organic matter on microbial euendolithic community composition and microbioerosion rates. Marine Ecol Progr Ser 392:1–15.

[obaa035-B8] Chazottes V, Le Campion-Alsumard T, Peyrot-Clausade M. 1995 Bioerosion rates on coral reefs: interactions between macroborers, microborers and grazers (Moorea, French Polynesia). Palaeogeogr Palaeoclimatol Palaeoecol 113:189–98.

[obaa035-B9] Che LM, Le Campion-Alsumard T, Boury-Esnault N, Payri C, Golubic S, Bézac C. 1996 Biodegradation of shells of the black pearl oyster, *Pinctada margaritifera var. cumingii*, by microborers and sponges of French Polynesia. Marine Biol 126:509–19.

[obaa035-B10] Clements KD, German DP, Piché J, Tribollet A, Choat JH. 2016 Integrating ecological roles and trophic diversification on coral reefs: multiple lines of evidence identify parrotfishes as microphages. Biol J Linnean Soc 120:729–51.

[obaa035-B11] Coker DJ, Graham NAJ, Pratchett MS. 2012 Interactive effects of live coral and structural complexity on the recruitment of reef fishes. Coral Reefs 31:919–27.

[obaa035-B12] Cook RD. 1977 Detection of influential observation in linear regression. Technometrics 19:15–8.

[obaa035-B13] Daval D, Guyot F, Bolotov IN, Vikhrev IV, Kondakov AV, Lyubas AA, Bychkov AY, Yapaskurt VO, Cabié M, Pokrovsky OS, et al 2020 Symbiotic cooperation between freshwater rock-boring bivalves and microorganisms promotes silicate bioerosion. Sci Rep 10:1–10. 32770130 10.1038/s41598-020-70265-x PMC7415154

[obaa035-B14] Davidson TM, Altieri AH, Ruiz GM, Torchin ME. 2018 Bioerosion in a changing world: a conceptual framework. Ecol Lett 21:422–38. 29314575 10.1111/ele.12899

[obaa035-B15] del Campo J, Pombert JF, Šlapeta J, Larkum A, Keeling PJ. 2017 The ‘other’ coral symbiont: ostreobium diversity and distribution. ISME J 11:296–9. 27420029 10.1038/ismej.2016.101 PMC5315466

[obaa035-B16] Fine M, Loya Y. 2002 Endolithic algae: an alternative source of photoassimilates during coral bleaching. Proc Royal Soc B 269:1205–10. 10.1098/rspb.2002.1983 PMC1691023 12065035

[obaa035-B17] Fine M, Meroz-Fine E, Hoegh-Guldberg O. 2005 Tolerance of endolithic algae to elevated temperature and light in the coral *Montipora monasteriata* from the southern Great Barrier Reef. J Exp Biol 208:75–81. 15601879 10.1242/jeb.01381

[obaa035-B18] Glynn PW, Manzello DP. 2015 Bioerosion and coral reef growth: a dynamic balance In: Birkeland C, editor. Coral reefs in the Anthropocene. New York (NY): Springer p 67–97.

[obaa035-B19] Graham NAJ, Nash KL. 2013 The importance of structural complexity in coral reef ecosystems. Coral Reefs 32:315–26.

[obaa035-B20] Grinham AR, Carruthers TJ, Fisher PL, Udy JW, Dennison WC. 2007 Accurately measuring the abundance of benthic microalgae in spatially variable habitats. Limnol Oceanogr Method 5:119–25.

[obaa035-B21] Jeffrey S. t, Humphrey G. 1975 New spectrophotometric equations for determining chlorophylls a, b, c1 and c2 in higher plants, algae and natural phytoplankton. Biochem Physiol Pflanzen 167:191–4.

[obaa035-B22] Jokiel PLM. 1978 Coral growth: buoyant weight technique In: Stoddart DR, Johannes RE, editors. Coral reef research methods. Paris, France: UNESCO p 529–42

[obaa035-B23] Kegler P, Kegler HF, Gärdes A, Ferse SCA, Lukman M, Alfiansah YR, Hassenrück C, Kunzmann A. 2017 Bacterial biofilm communities and coral larvae settlement at different levels of anthropogenic impact in the Spermonde Archipelago, Indonesia. Front Marine Sci 4:270.

[obaa035-B24] Kleemann K. 1980 Boring bivalves and their host corals from the Great Barrier Reef. J Mollus Stud 46:13–54.

[obaa035-B25] Kleemann K. 1996 Biocorrosion by bivalves. Marine Ecol 17:145–58.

[obaa035-B26] Kühl M, Holst G, Larkum AW, Ralph PJ. 2008 Imaging of oxygen dynamics within the endolithic algal community of the massive coral *Porites lobata*. J Phycol 44:541–50. 27041414 10.1111/j.1529-8817.2008.00506.x

[obaa035-B27] Larkum AW, Kühl M. 2005 Chlorophyll d: the puzzle resolved. Trend Plant Sci 10:355–7. 10.1016/j.tplants.2005.06.005 16019251

[obaa035-B28] Marcelino VR, Verbruggen H. 2016 Multi-marker metabarcoding of coral skeletons reveals a rich microbiome and diverse evolutionary origins of endolithic algae. Sci Rep 6:31508. 27545322 10.1038/srep31508 PMC4992875

[obaa035-B29] Massé A, Domart-Coulon I, Golubic S, Duché D, Tribollet A. 2018 Early skeletal colonization of the coral holobiont by the microboring Ulvophyceae Ostreobium sp. Sci Rep 8:11. 29396559 10.1038/s41598-018-20196-5 PMC5797222

[obaa035-B30] Miyajima T, Suzumura M, Umezawa Y, Koike I. 2001 Microbiological nitrogen transformation in carbonate sediments of a coral-reef lagoon and associated seagrass beds. Marine Ecol Prog Ser 217:273–86.

[obaa035-B31] Mokady O, Loya Y, Lazar B. 1998 Ammonium contribution from boring bivalves to their coral host–a mutualistic symbiosis? Marine Ecol Prog Ser 169:295–301.

[obaa035-B32] Pernice M, Raina JB, Rädecker N, Cárdenas A, Pogoreutz C, Voolstra CR. 2020 Down to the bone: the role of overlooked endolithic microbiomes in reef coral health. ISME J 14:325–10. 31690886 10.1038/s41396-019-0548-z PMC6976677

[obaa035-B33] Perry CT, Murphy GN, Kench PS, Edinger EN, Smithers SG, Steneck RS, Mumby PJ. 2014 Changing dynamics of Caribbean reef carbonate budgets: emergence of reef bioeroders as critical controls on present and future reef growth potential. Proc Royal Soc B 281:20142018. 10.1098/rspb.2014.2018 PMC4213658 25320166

[obaa035-B34] R Core Team. 2019 R: a language and environment for statistical computing. Vienna, Austria: R Foundation for Statistical Computing.

[obaa035-B35] Reusch TB, Chapman AR, Gröger JP. 1994 Blue mussels *Mytilus edulis* do not interfere with eelgrass Zostera marina but fertilize shoot growth through biodeposition. Marine Ecol Prog Ser 108:265–82.

[obaa035-B36] Reyes-Nivia C, Diaz-Pulido G, Kline D, Guldberg OH, Dove S. 2013 Ocean acidification and warming scenarios increase microbioerosion of coral skeletons. Global Change Biol 19:1919–29. 10.1111/gcb.12158 23505093

[obaa035-B37] Ricci F, Marcelino VR, Blackall LL, Kühl M, Medina M, Verbruggen H. 2019 Beneath the surface: community assembly and functions of the coral skeleton microbiome. Microbiome 7:159. 31831078 10.1186/s40168-019-0762-y PMC6909473

[obaa035-B38] Rice MM, Maher RL, Correa AMS, Moeller HV, Lemoine NP, Shantz AA, Burkepile DE, Silbiger NJ. 2020 Macroborer presence on corals increases with nutrient input and promotes parrotfish bioerosion. Coral Reefs 39:409–18.

[obaa035-B39] Ritchie R. 2008 Universal chlorophyll equations for estimating chlorophylls a, b, c, and d and total chlorophylls in natural assemblages of photosynthetic organisms using acetone, methanol, or ethanol solvents. Photosynthetica 46:115–26.

[obaa035-B40] Roff G, Joseph J, Mumby PJ. 2019 Multi-decadal changes in structural complexity following mass coral mortality on a Caribbean reef. Biogeosciences Discussions 1–16.

[obaa035-B41] Rotjan RD, Lewis SM. 2005 Selective predation by parrotfishes on the reef coral *Porites astreoides*. Marine Ecol Prog Ser 305:193–201.

[obaa035-B42] Sangsawang L, Casareto BE, Ohba H, Vu HM, Meekaew A, Suzuki T, Yeemin T, Suzuki Y. 2017 ^13^C and ^15^N assimilation and organic matter translocation by the endolithic community in the massive coral *Porites lutea*. Royal Soc Open Sci 4:171201. 10.1098/rsos.171201 PMC5750018 29308251

[obaa035-B43] Santos IR, Erler D, Tait D, Eyre BD. 2010 Breathing of a coral cay: tracing tidally driven seawater recirculation in permeable coral reef sediments. J Geophys Res Oceans 115:C12010.

[obaa035-B44] Schlichter D, Zscharnack B, Krisch H. 1995 Transfer of photoassimilates from endolithic algae to coral tissue. Naturwissenschaften 82:561–4.

[obaa035-B45] Schönberg CH, Fang JK, Carreiro-Silva M, Tribollet A, Wisshak M. 2017 Bioerosion: the other ocean acidification problem. ICES J Marine Sci 74:895–925.

[obaa035-B46] Scott P, Risk MJ. 1988 The effect of *Lithophaga* (Bivalvia: Mytilidae) boreholes on the strength of the coral Porites lobata. Coral Reefs 7:145–51.

[obaa035-B47] Simon-Blecher N, Achituv Y, Malik Z. 1996 Effect of epibionts on the microdistribution of chlorophyll in corals and its detection by fluorescence spectral imaging. Marine Biol 126:757–63.

[obaa035-B48] Tribollet A, Golubic S. 2005 Cross-shelf differences in the pattern and pace of bioerosion of experimental carbonate substrates exposed for 3 years on the northern Great Barrier Reef. Austral Coral Reefs 24:422–34.

[obaa035-B49] Vergés A, Vanderklift MA, Doropoulos C, Hyndes GA. 2011 Spatial patterns in herbivory on a coral reef are influenced by structural complexity but not by algal traits. PLoS One 6:e17115. 21347254 10.1371/journal.pone.0017115 PMC3037963

[obaa035-B50] Veron JEN, Stafford-Smith M. 2000 Corals of the world. Queensland, Australia: Townsville MC.

